# Bilateral Purtscher-like retinopathy: A case report in a patient with pre-eclampsia and post intrauterine fetal demise

**DOI:** 10.1016/j.ijscr.2023.109072

**Published:** 2023-11-23

**Authors:** Nora Aldhefeery, Danah Aldhafiri, Mohamed Fathy, Shyji Kumaran, Mohamed Abdelbadie

**Affiliations:** a Department of Ophthalmology, Farwaniya Hospital, Ministry of Health, Kuwait; b Faculty of Medicine, Kuwait University, Kuwait

**Keywords:** Purtscher-like retinopathy, Purtscher's retinopathy, Preeclampsia

## Abstract

**Introduction:**

Purtscher's retinopathy is a rare ophthalmic condition with unclear pathogenesis commonly related to trauma and affecting young or middle-aged men. The annual incidence in the UK has been estimated to be 0.24 cases per million.

**Presentation of case:**

A 29-year-old primigravida female, previously healthy with no antenatal care, was brought to the maternal causality at 36 weeks of gestation as a case of pre-eclampsia and intrauterine fetal demise (IUFD) after a prolonged delivery attempt at home. After delivery, the patient reported bilateral vision loss. On ophthalmic examination, the best-corrected visual acuity (BCVA) was count fingers at 15 cm in both eyes. Dilated fundus exam showed scattered flame-shaped hemorrhage, multiple cotton wool Spots known as Purtscher flecken were seen in the distribution of the radial peripapillary capillary, dot and blot hemorrhage, and macular star in both eyes. The patient was treated with labetalol and magnesium sulphate for systemic control of arterial blood pressure by the obstetrics team, and managed conservatively under ophthalmic observation. Upon five months follow-up, BCVA had improved bilaterally.

**Discussion:**

Visual changes have been reported in 25 % of patients with preeclampsia. Although most of these changes are transient, some rare sight-threatening eye conditions have been linked to preeclampsia namely Purtscher-like retinopathy.

**Conclusions:**

Purtscher-like retinopathy is rarely reported in preeclampsia and after childbirth. Although most of visual changes associated with preeclampsia are transient, urgent ophthalmology consultation is important to rule out serious etiologies such as Purtscher-like retinopathy.

## Highlights

• Visual changes in patients with preeclampsia should be evaluated to rule out purtscher-like retinopathy
• Preeclampsia and valsalva maneuver technique during the second stage of labor may contribute to developing purtscher-like retinopathy
• Purtscher-like retinopathy is rarely reported in preeclampsia and after childbirth

## 1 Introduction

Purtscher's retinopathy is an occlusive microvasculopathy characterized by multiple retinal white areas around the optic nerve head and fovea with paravascular clearing, which may be associated with intraretinal hemorrhages [1]. It is considered a rare sight-threatening ophthalmic condition, commonly affecting young or middle-aged men [2]. Typical changes seen in Purtscher's retinopathy include cotton wool spots, Purtscher flecken, and minimal intraretinal hemorrhage [3].

Purtscher's retinopathy is usually caused by trauma, particularly cranial trauma or thoracic compression. However, when the etiology is non-traumatic in nature, the term “Purtscher-like retinopathy” is used [4]. Non-traumatic conditions that are associated with Purtscher-like retinopathy include Acute pancreatitis, vasculitis, renal failure, and systemic lupus erythematous. Although rare, it has also been reported postpartum in patients with preeclampsia and HELLP syndrome [4,5]. In this report, we present a rare case of Purtscher-like retinopathy after a prolonged delivery attempt at home in a patient with pre-eclampsia and intrauterine fetal demise (IUFD).

This case report has been reported in line with SCARE Criteria [6].

## 2 Presentation of case

A 29-year-old primigravida female, previously healthy with no antenatal care, was brought to the maternal causality by an ambulance at 36 weeks of gestation as a case of pre-eclampsia and intrauterine fetal demise (IUFD) after a prolonged delivery attempt at home. The patient arrived with the head of a dead macerated fetus outside the vagina. The baby and the placenta were completely delivered via assisted vaginal delivery.

Her vitals on admission were as follows: blood pressure was 190/118, heart rate 140 beats per minute, temperature 36.8 °C, and oxygen saturation 98 %. Her urine protein was 3+ and 24-hour urinary protein excretion was 1.04 g/24 h.

**Fig. 1:**
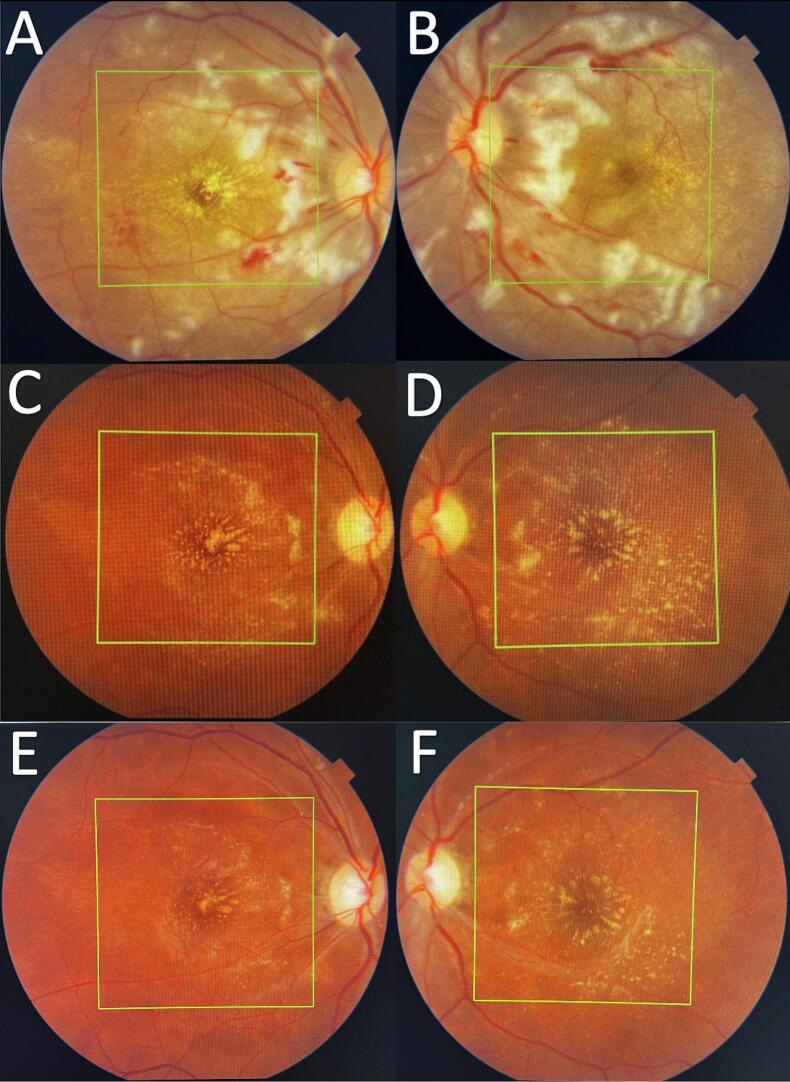
[**a,b.** Left eye and right eye fundus photos showed scattered flame-shaped hemorrhage, multiple cotton wool Spots known as Purtscher flecken are seen in the distribution of the radial peripapillary capillary, dot and blot hemorrhage, and macular star in both eyes. **C,d.** Left eye and right eye fundus photos showed resolution of retinal whitening and hemorrhages without any other acute lesions. **E,f.** Left eye and right eye fundus photos showed optic disc pallor, Sheathing of retinal vessels, sclerosed retinal arteries, resolved retinal cotton wool spots, and hemorrhages without any acute lesion].

**Fig. 2:**
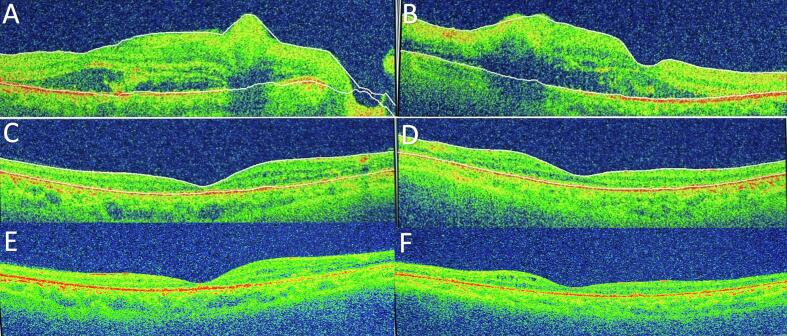
[**a,b.** Optical coherence tomography (OCT) showed a homogeneous hyper-reflective nerve fiber layer as a result of ischemic edema of nervous layer, and severe central macular edema with the presence of serous subretinal fluid causing sub-foveal serous macular neurosensory detachment **C,d,e,f.** OCT revealed a complete resolution of macular edema and neurosensory detachment with recovery of foveal depression; discrete thinning and disorganization of the inner retinal layers but preserved photoreceptor's layer.]

Three hours after delivery, the patient complained of bilateral painless blurred vision and headache. The patient denied any flashes, floaters, eye pain, ocular, or extra-ocular trauma. On ophthalmic examination, the best-corrected visual acuity (BCVA) was close to the face counting fingers in both eyes. Anterior segment examination was within normal limits in both eyes. Pupillary light reflexes, intraocular pressure (IOP), and extraocular eye movement (EOM) all were normal. A dilated fundus exam showed a normal clear vitreous, normal optic disc, scattered flame-shaped hemorrhage, multiple cotton wool Spots known as Purtscher flecken were seen in the distribution of the radial peripapillary capillary, dot and blot hemorrhage, and macular star in both eyes (Fig. 1a, b). In addition, there was a retinal choroidopathy with serous macular neurosensory detachment. Optical coherence tomography (OCT) showed a homogeneous hyper-reflective nerve fiber layer as a result of ischemic edema of nervous layer, and severe central macular edema with the presence of serous subretinal fluid causing sub-foveal serous macular neurosensory detachment (Fig. 2a, b).

Based on clinical presentation and ophthalmic examination the patient was diagnosed with Purtscher-like retinopathy with hypertensive chorioretinopathy.

All the following laboratory tests were within normal ranges: complete blood count, renal function test, liver function test, and coagulation profile. Infectious diseases screening (HBsAg, HCV, HIV, and VDRL test) and autoimmune markers were both unremarkable as well. CT head angiography showed small areas of low attenuation at both insular cortexes more prominent on the left side likely sub-acute ischemic changes.

Since there is no clear treatment guideline for Purtscher-like retinopathy, the patient was managed by the obstetrics team with labetalol and magnesium sulphate to control the blood pressure and managed conservatively under ophthalmic observation. Two weeks after the admission, the patient reported improvement in vision; however, there were no changes in visual acuity in both eyes. Fundus exam revealed a progressive reduction in size and number of cotton-wool spots and Purtscher flecken. After the patient was medically stable, she was discharged from the hospital with an ophthalmology follow-up after two months.

Two months later, the BCVA for both eyes was still counting fingers. Fundoscopy showed resolution of retinal whitening and hemorrhages without any other acute lesions (Fig. 1c, d). OCT revealed a complete resolution of macular edema and neurosensory detachment with recovery of foveal depression; discrete thinning and disorganization of the inner retinal layers but preserved photoreceptor's layer (Fig. 2c, d).

**Fig. 3:**
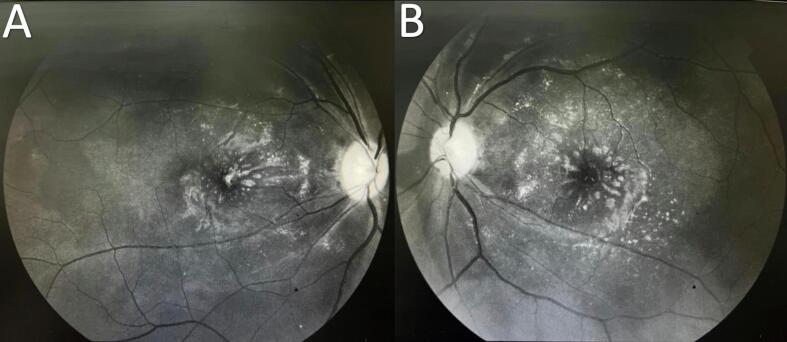
[**a,b.** Red free fundus images showed optic disc pallor, sheathing of retinal vessels, sclerosed retinal arteries, resolved retinal cotton wool spots, and hemorrhages].

On five-month follow-up, the BCVA was 20/40p in the right eye and 20/50p in the left eye. The color vision was tested with Ishihara test, and she was able to read only 1 out of 13 plates. The visual field showed concentric contraction. Fundoscopy and red free fundus images showed optic disc pallor, sheathing of retinal vessels, sclerosed retinal arteries, resolved retinal cotton wool spots, and hemorrhages without any acute lesion (Fig. 1e, f) (Fig. 3a, b). OCT showed a complete resolution of macular edema and neurosensory detachment with recovery of foveal depression; discrete thinning and disorganization of the inner retinal layers but preserved photoreceptor's layer (Fig. 2e, f).

## 3 Discussion

Purtscher's retinopathy is a rare ophthalmic condition with unclear pathogenesis [7,8]. The annual incidence in the UK has been estimated to be 0.24 cases per million [7]. Patients with Purtscher-like retinopathy usually complain of bilateral but asymmetrical vision loss that can range from a minor loss to hand motion acuity [9,10]. Cotton wool spots and retinal hemorrhage are the most common findings associated with this condition. Approximately 50 % of patients with Purtscher-like retinopathy presented with a pathognomonic sign known as Purtscher flecken [11].

Visual changes have been reported in 25 % of patients with preeclampsia. Although most of these changes are transient, some rare sight-threatening eye conditions have been linked to preeclampsia namely Purtscher-like retinopathy [7,12,13]. Patients with Purtscher-like retinopathy usually present within the first 48 h postpartum [14]. Similarly, in our case, vision loss was reported 3 h postpartum. The pathophysiology of pre-eclampsia is not yet known but is thought to include reduced organ perfusion and endothelial dysfunction [15]. Microvascular circulation can be compromised in preeclampsia affecting cerebral and ocular function [12]. Even though CT head angiography in our patient showed a small area of low attenuation at both insular cortexes suggesting subacute ischemic changes, the cerebral function was intact.

Multiple reports showed that Purtscher-like retinopathy may develop after extreme valsalva, such as weightlifting [16]. It is important to note that a valsalva maneuver technique is frequently used during the second stage of labor [17]. Our patient had a prolonged delivery attempt at home without any medical assistance before coming to the hospital, which we think might have played a role in her presentation.

Although there is no clear guideline to diagnose or treat Purtscher-like retinopathy [18]. Miguel et al. introduced the first diagnostic criteria which include the presence of Purtscher flecken, the presence of flame-shaped or dot-and-blot retinal hemorrhages, the presence of cotton-wool spots, reasonable etiology, and compatible complementary investigation. They suggested that a diagnosis of Purtscher-like retinopathy is established when at least three of the diagnostic criteria are met, and the retinal findings are limited to the posterior wall with no history of ocular trauma [19]. Successful treatment with high-dose steroids in intravitreal form has been reported in multiple cases; however, the recommended treatment modality is conservative follow-up [20].

The prognosis of Purtscher-like retinopathy depends on the disease severity at presentation. In around 50 % of cases, the vision improves spontaneously more than 2 Snellen lines [7]. Our patient did not show any progress in the 2-month follow-up, but in the 5-month follow-up, the BCVA was 20/40 p and 20/50 p in the right and left eyes, respectively with abnormal color vision. In addition, complications that can manifest in patients with Purtscher-like retinopathy include optic atrophy, mottling of the retinal pigmented epithelium, retinal thinning, and narrowing of retinal arteries [21]. Likewise, our patient's fundoscopy exam showed optic atrophy and a sclerosed retinal artery.

## 4 Conclusions

Purtscher-like retinopathy is a sight-threatening eye condition that is rarely reported in preeclampsia and after childbirth. Although most of visual changes associated with preeclampsia are transient, urgent ophthalmology consultation is important to rule out serious etiologies such as Purtscher-like retinopathy.

## Ethical approval

The study is exempt from ethical approval in our Hospital in Kuwait.

## Funding

This research did not receive any specific grant from funding agencies in the public, commercial, or not-for-profit sectors.

## Author contribution

Nora A Aldhefeery data collection, data analysis, interpretation, writing the paper.

Danah Aldhafiri data analysis, interpretation, writing the paper.

Mohamed Fathy study concept, design, and review

Shyji Kumaran data collection, data analysis, interpretation.

Mohamed Abdelbadie study concept, design, and review

## Guarantor

Nora Aldhefeery.

## Patient consent

Written informed consent was obtained from the patient for publication of this Case Report and any accompanying images. A copy of the written consent is available for review by the Editor of this journal.

## Authorship

All authors attest that they meet the current ICMJE criteria for Authorship.

## Declaration of competing interest

All authors have no financial disclosures.
